# Investigation into the effect of deltoid ligament injury on rotational ankle instability using a three-dimensional ankle finite element model

**DOI:** 10.3389/fbioe.2024.1386401

**Published:** 2024-05-01

**Authors:** Yuandong Li, Jiahui Tong, Huizhi Wang, Xiaoxi Ji, Yinghui Hua, Cheng-Kung Cheng

**Affiliations:** ^1^ School of Biomedical Engineering, Shanghai Jiao Tong University, Engineering Research Center for Digital Medicine of the Ministry of Education, Shanghai, China; ^2^ Department of Sports Medicine, Huashan Hospital, Shanghai, China; ^3^ Center for Intelligent Medical Equipment and Devices, Institute for Innovative Medical Devices, University of Science and Technology of China, Hefei, China; ^4^ Suzhou Institute for Advanced Research, University of Science and Technology of China, Suzhou, China

**Keywords:** finite element, rotational ankle instability, deltoid ligament, anterior talofibular ligament finite element, anterior talofibular ligament

## Abstract

**Background:**

Injury to the lateral collateral ligament of the ankle may cause ankle instability and, when combined with deltoid ligament (DL) injury, may lead to a more complex situation known as rotational ankle instability (RAI). It is unclear how DL rupture interferes with the mechanical function of an ankle joint with RAI.

**Purpose:**

To study the influence of DL injury on the biomechanical function of the ankle joint.

**Methods:**

A comprehensive finite element model of an ankle joint, incorporating detailed ligaments, was developed from MRI scans of an adult female. A range of ligament injury scenarios were simulated in the ankle joint model, which was then subjected to a static standing load of 300 N and a 1.5 Nm internal and external rotation torque. The analysis focused on comparing the distribution and peak values of von Mises stress in the articular cartilages of both the tibia and talus and measuring the talus rotation angle and contact area of the talocrural joint.

**Results:**

The dimensions and location of insertion points of ligaments in the finite element ankle model were adopted from previous anatomical research and dissection studies. The anterior drawer distance in the finite element model was within 6.5% of the anatomical range, and the talus tilt angle was within 3% of anatomical results. During static standing, a combined rupture of the anterior talofibular ligament (ATFL) and anterior tibiotalar ligament (ATTL) generates new stress concentrations on the talus cartilage, which markedly increases the joint contact area and stress on the cartilage. During static standing with external rotation, the anterior talofibular ligament and anterior tibiotalar ligament ruptured the ankle’s rotational angle by 21.8% compared to an intact joint. In contrast, static standing with internal rotation led to a similar increase in stress and a nearly 2.5 times increase in the talus rotational angle.

**Conclusion:**

Injury to the DL altered the stress distribution in the tibiotalar joint and increased the talus rotation angle when subjected to a rotational torque, which may increase the risk of RAI. When treating RAI, it is essential to address not only multi-band DL injuries but also single-band deep DL injuries, especially those affecting the ATTL.

## Introduction

Ankle instability, commonly seen in athletes, is often attributed to injury of the anterior talofibular ligament (ATFL) (Hintermann et al., 2002). Instability can hamper the patient’s ability to engage in physical activities and result in abnormal joint function, increasing the risk of osteoarthritis (Golditz et al., 2014). Orthopedic surgeons recognize that combined injury to the ATFL and deltoid ligament (DL) can lead to a more complicated condition known as Rotational Ankle Instability (RAI) (Valderrabano et al., 2007). Studies have shown that in cases of ATFL injury, there is a 40% likelihood of concurrent deltoid ligament injury (Koh et al., 2023). The prevailing treatment for RAI in clinical practice focuses on repairing or reconstructing the lateral ATFL and calcaneofibular ligament (CFL). However, postoperative follow-up studies have reported that up to 30% of patients are dissatisfied with the results (Harper, 1988; Vega et al., 2020), and around 60% continue to experience tenderness in the medial ankle (Yang et al., 2023).

The high dissatisfaction rate may be due to inadequate treatment of the damage to the medial ligament, suggesting a need for more comprehensive approaches to treating RAI (Hintermann, 2003; Hintermann et al., 2004). A similar lack of consensus exists on the best strategy for treating DL injuries. Vega et al. introduced a surgical technique for repairing the anterior fibers of the deltoid ligament using a single anchor and non-absorbable sutures (Vega et al., 2020), and Li et al. detailed a method for repairing the deep fibers in the DL using a similar approach (Li et al., 2023). Higashiyama et al. used autologous tendon grafts for anterior tibiotalar ligament (ATTL) reconstruction (Higashiyama et al., 2020). Choi et al. developed a process using three anchors and suture tape to repair the superficial layer of the DL (Choi et al., 2016). The variation in repair methods may be due to the limited understanding of the underlying mechanisms of RAI (Higashiyama et al., 2020; Vega et al., 2020; Mansur et al., 2021; Pisanu et al., 2021). A more thorough understanding of the mechanical contribution of the individual ligaments to ankle joint stability is essential for developing effective methods for treating RAI (Li et al., 2019). Research in this field often uses a combination of biomechanical testing of cadaveric ligaments alongside finite element simulation for analytical purposes.

To assess the effects of various ligament injuries on ankle stability, researchers typically subject cadaveric foot and ankle specimens to a range of simulated injuries. These specimens are then subjected to forces and torques on a stress platform, while X-ray images record changes in ankle joint displacement or the talus rotational angle (Tarczyńska et al., 2020; Teramoto et al., 2021; Bhimani et al., 2022; Saengsin et al., 2022). Previous research indicated that the deltoid ligament complex stabilizes the ankle joint’s medial aspect, limiting hindfoot eversion and talus external rotation (Cromeens et al., 2015; Brodell et al., 2019). Nonetheless, few studies have assessed in detail the biomechanical contribution of each band of the deltoid ligament to ankle stability (Wei et al., 2011; Hsu et al., 2015; Takao et al., 2020). While cadaveric specimens allow for *in vivo* testing in human tissue, this method has considerable drawbacks, such as specimen availability, formalin-induced tissue changes, and limitations in measuring internal stress in the joint. The aforementioned limitations and variability in experimental methodologies across studies lead to substantial variation between results. Finite Element Analysis (FEA) has emerged as a complementary tool for biomechanical testing (Park et al., 2019; Alastuey-López et al., 2021; Wang et al., 2022; Luan et al., 2023). FEA allows for individual variables to be controlled, provides a broader range of test conditions compared to cadaveric experiments, is not limited by sample availability, and is not subject to the same ethical considerations (Zhang et al., 2011; Xu et al., 2012). Moreover, it allows stress and deformation behaviors to be predicted in deep internal tissues (Cheung et al., 2005; Wong et al., 2016) and has been used to compare treatment methods and develop innovative strategies that may not be possible using conventional test methods (Isvilanonda et al., 2012). Several finite element models of the ankle ligament complex have been proposed to investigate limitations with current clinical treatments (Cheung et al., 2006; Tao et al., 2009; Shin et al., 2012; Mo et al., 2022). However, these previous models have limited applications because injury to the medial deltoid ligament is often overlooked (Can et al., 2020), uniform stiffness is usually assigned across all ligaments, and the ligaments are frequently oversimplified or incomplete (Forestiero et al., 2017). Such shortcomings can lead to considerable variability in the outcomes and undermine the accuracy of the results.

As such, this study aims to 1) develop a comprehensive finite element numerical model of the foot and ankle, incorporating a complete range of ligaments. The ligaments were constructed as solid models from MRI images with realistic anatomical structures, facilitating a better understanding of the internal behavior of the ligaments under load. The interface between the ligament and surrounding tissues can be more accurately simulated, thus providing a robust tool for analyzing ankle mechanics under various conditions, and 2) examining the talus rotation and stress distribution in the tibiotalar joint surface cartilage in cases of combined medial and lateral ligament injuries. It was hypothesized that there is considerable variation in the rotational stability of the ankle joint with different ligament injuries, and the damage to the deep layer of the deltoid ligament, compared to the superficial layer, has a more significant effect on the rotational stability of the ankle joint, leading to abnormal joint stress and contact patterns. Clearly defining the mechanical contributions and biomechanical responses of each ligament to the rotational stability of the ankle joint is essential for pre-operative planning and choosing the correct approach for repair and reconstruction.

## Materials and methods

This study used a finite element model of the right ankle to simulate a static stance and internal-external rotation tests under various ligament rupture conditions: (1) an ankle with intact ligaments; (2) isolated ATFL rupture; (3) ATFL rupture associated with various combinations of superficial deltoid ligament injuries, including talonavicular ligament (TNL), tibiospring ligament (TSL), tibiocalcaneal ligament (TCL), and superficial posterior tibiotalar ligament (sPTTL); (4) ATFL rupture with different combinations of deep deltoid ligament injuries, comprising anterior tibiotalar ligament (ATTL) and deep posterior tibiotalar ligament (dPTTL) ruptures; (5) ruptures of all four superficial bundles of the deltoid ligament; (6) ATFL rupture accompanied by ruptures of all four superficial bundles of the deltoid ligament. The analysis focused on ankle kinematics, maximum von Mises stress on tibial and talus articular cartilage, joint compressive contact area, and the talus rotation angle.

### Model establishment

**FIGURE 1:**
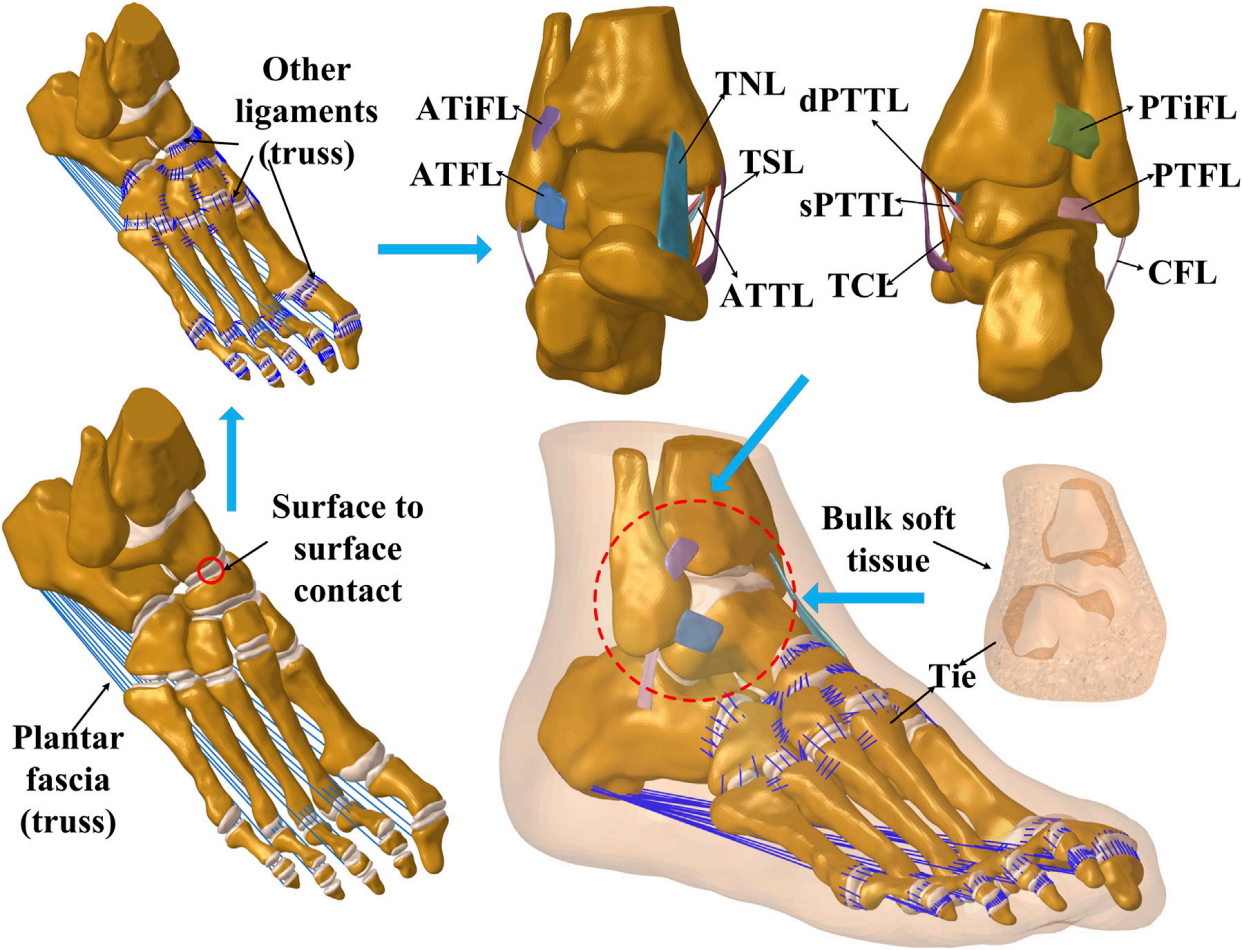
Overview of the finite element foot-ankle model showing bones, tissues and connections.

A comprehensive three-dimensional foot-ankle model was constructed from MR images of an adult female right foot specimen (162 cm, 57 kg) sourced from Huashan Hospital, affiliated with Fudan University. Consent for the storage and utilization of the bodies for research was obtained from the donors before their death or their next of kin. The parameters are as follows: TE = 4.3, TR = 380, resolution = 0.4 × 0.4 mm, and slice thickness = 0.4 mm. The MR images were reconstructed in Mimics 21.0 (Materialise N. V., Leuven, Belgium), followed by surface refinement and feature extraction in Geomagic Studio (Geomagic, version 12.0). The model was then meshed using HyperMesh (Altair Engineering, Tokyo, Japan) and imported into Abaqus (Simulia, Inc, USA, version 2016) for preprocessing, finite element analysis, and subsequent post-processing. The final FE model encompassed 26 complete foot bones, including the distal ends of the tibia and fibula. The cartilage of each joint in the ankle was meticulously segmented and included in the model. The plantar fascia and several small ligaments dispersed throughout the foot were modeled with tension-only truss elements linked to their respective bone attachment points. The model also simulated eleven primary ligaments surrounding the ankle joint, with five laterally (ATFL, posterior talofibular ligament (PTFL), CFL, anterior tibiofibular ligament (ATiFL), posterior tibiofibular ligament (PTiFL)) and six medially (TSL, TCL, TNL, ATTL, dPTTL, sPTTL). Experienced ankle surgeons rigorously verified all ligament segmentations and insertion points. The final three-dimensional finite element ankle model is shown in Figure 1. As detailed in Table 1, the material parameters of tissues and element types were adopted from the literature. Cartilage-to-cartilage interactions were defined as frictionless sliding contacts, permitting mutual sliding without penetration. On the other hand, ligament insertion points were bound to their respective bone insertion points to maintain consistent displacement. Binding constraints were also applied between cartilage and the adjoining bone surface.

**TABLE 1:** Summary of model materials and element types.

Component		Element	Material types	Young’s modulus (MPa)	Poisson’s ratio	References
Bulk soft tissue		Tetrahedra	Linearly elastic	0.15	0.45	Cheung and Zhang (2005)
Bone		Tetrahedra	Linearly elastic	7,300	0.3	Nakamura et al. (1981)
Cartilage		Tetrahedra	Linearly elastic	45	0.42	Dakin et al. (2001)
Ankle Ligaments	ATFL	Tetrahedra	Linearly elastic	255.5	0.4	Siegler et al. (1988), Li et al. (2020)
	CFL	Tetrahedra	Linearly elastic	512	0.4	
	PTFL	Tetrahedra	Linearly elastic	216.5	0.4	
	ATiFL	Tetrahedra	Linearly elastic	260	0.4	
	PTiFL	Tetrahedra	Linearly elastic	260	0.4	
	TNL	Tetrahedra	Linearly elastic	320.7	0.4	
	TSL	Tetrahedra	Linearly elastic	184.5	0.4	
	CTL	Tetrahedra	Linearly elastic	512	0.4	
	ATTL	Tetrahedra	Linearly elastic	184.5	0.4	
	sPTTL	Tetrahedra	Linearly elastic	99.5	0.4	
	dPTTL	Tetrahedra	Linearly elastic	99.5	0.4	
Plantar fascia		Tension- only truss	Linearly elastic	350	0.35	Cheung and Zhang (2005)
Other ligaments		Tension- only truss	Linearly elastic	260	0.35	Yu et al. (2013)

Mesh convergence testing was executed by applying an anterior drawer force of 150 N to the ankle joint and calculating the distance between the posterior edges of the tibia and talus. The selected mesh used first-order, four-node tetrahedron elements. The mesh size was incrementally decreased until the variation in the specified distance remained within 2% with the reduction in element size. The final element size for ligaments and cartilage was 0.5 mm, while other structures were set at 1 mm. The entire model comprised 1,739,585 elements.

### Validation of the FE model

**FIGURE 2:**
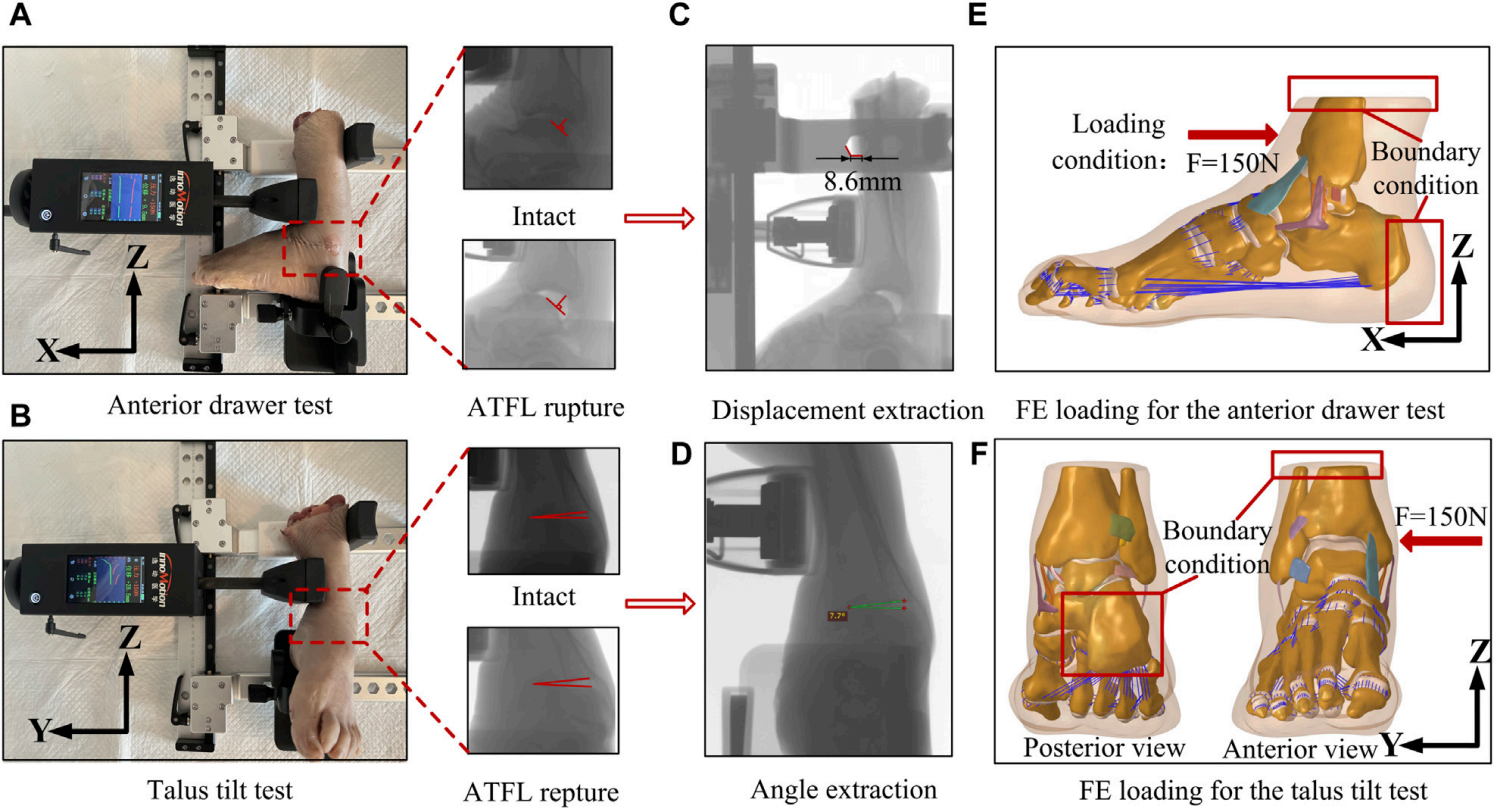
Biomechanical experiments on ankle specimens and the finite element model setup using the same boundary and loading conditions. **(A)** Diagram of anterior drawer test. **(B)** Diagram of talus tilt test. **(C)** Extraction of anterior drawer displacement with X-ray platform. **(D)** Extraction of talus tilt angle with X-ray platform. **(E)** Boundary conditions and loading for finite element simulation of anterior drawer test. **(F)** Boundary conditions and loading for finite element simulation of talus tilt test.

For biomechanical validation, a Ligs digital joint meter (Innomotion Inc., China) and an X-ray imaging system were used to perform an anterior drawer test (ADT) and a talus tilt test on the ankle specimen. This allowed for objective measurements of the anterior drawer test distance and the talus tilt angle. The left foot sample was used in this experiment, and the right foot finite element model originated from the same individual. As shown in Figures 2A,B, during the anterior drawer test, the device’s motor unit methodically retracted the anterior tibia relative to a static calf and heel, applying a force of up to 150 N at 3 N/s. X-ray images were taken for further analysis after the ankle specimen was subjected to a 150 N anterior drawer force. Subsequently, the apparatus was adjusted to apply a force of the same magnitude and loading rate to the medial side of the tibia, and X-ray images were recorded. Then, an ankle surgeon dissected the specimen, severed the ATFL, and repeated the two experiments mentioned above. Figures 2C,D, respectively, demonstrate the method of extracting the vertical distance between the posterior edges of the tibia and talus, known as the anterior drawer distance, and the talus tilt angle from X-ray images using the RadiAnt DICOM Viewer software. Each measurement was taken three times and the average was considered the final value. Figures 2E,F show the setup method for the finite element model, which adheres to the same boundary and loading conditions used in the biomechanical experiments. In the simulated anterior drawer test, the heel end was fixed, and the distal end of the tibia and fibula was constrained to allow only rotational movement around the *Y*-axis. Then, a 150 N force was applied at the corresponding position on the anterior edge of the tibia, and the vertical distance between the posterior edges of the tibia and talus was extracted in the post-processing of the model. In the simulated talus tilt test, the model’s heel was fixed, and the distal end of the tibia and fibula was constrained to allow only rotational movement around the *x*-axis. Then, a 150 N force was applied to the corresponding position on the medial side of the tibia, and the rotational angle of the talus was extracted in the post-processing. Ultimately, the finite element simulation results were compared with the experimental data to determine the model’s accuracy.

The skin, fat, fascia, muscles, and anterior and lateral tendons of the ankle joint were meticulously excised to unveil the ligaments underneath for anatomical validation. With the ankle joint neutrally positioned, a vernier caliper (accurate to 0.1 mm) was used to record the length of each ligament’s upper and lower boundaries. The ligament width and thickness were determined at three locales: proximal, distal, and midpoint. Given the bifurcation of the medial deltoid ligament, post superficial measurement, it was gently separated to inspect its deeper segments. Furthermore, the positions of the ligament insertion in the model were corroborated using measurements from a three-dimensional coordinate measuring device, as detailed by Campbell et al., which assessed the distances from the insertion of each ligament to pertinent bone landmarks (Campbell et al., 2014).

### Establishment and simulation of intact and injury models

**FIGURE 3:**
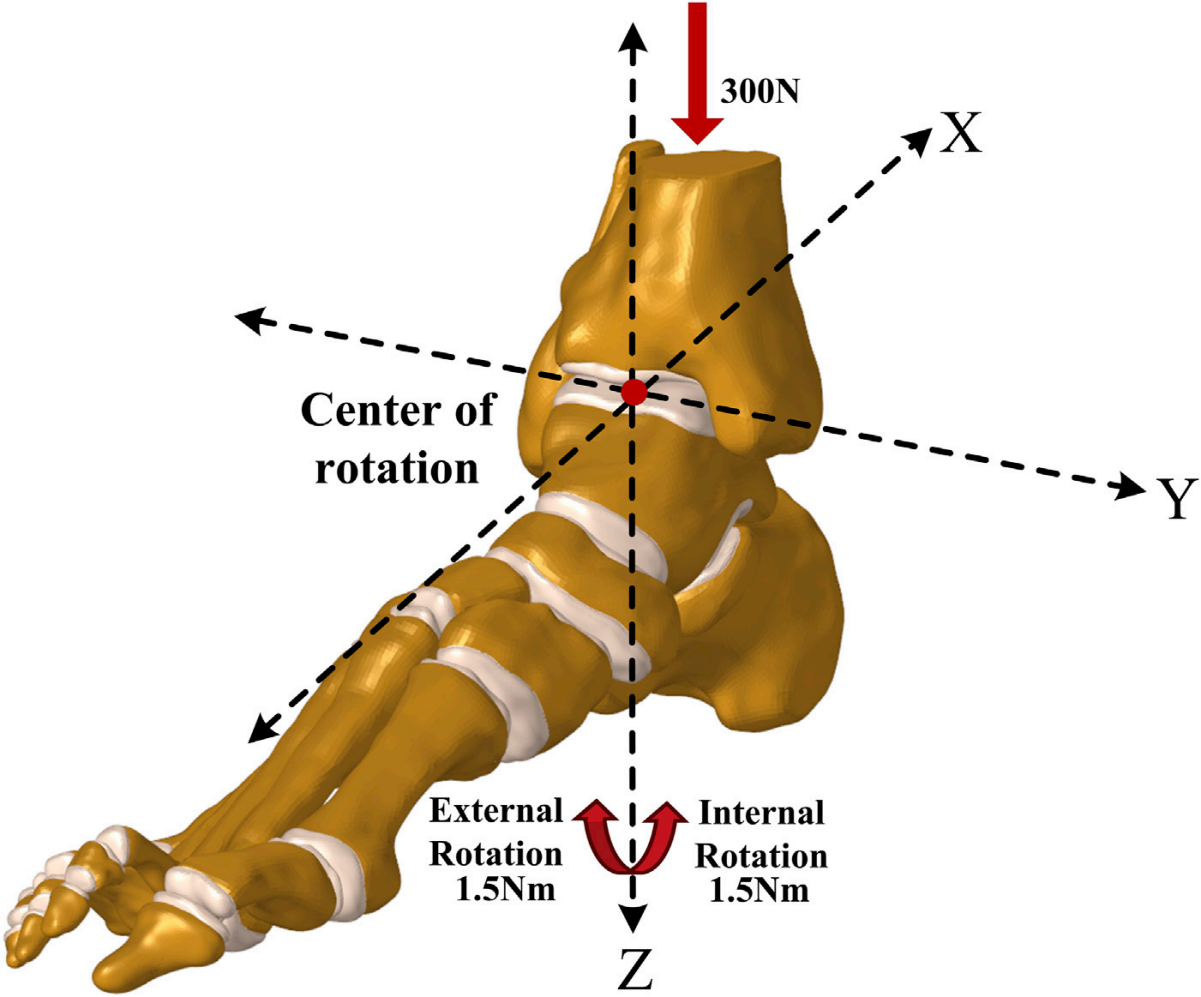
The setup of finite element models using identical boundary and loading conditions.

The coordinate system for the rotation axis in the finite element model was defined using a method introduced by Brockett and Chapman (Brockett and Chapman, 2016), as shown in Figure 3. In this system, the *Y*-axis of the ankle joint complex on the sagittal plane is defined by a line connecting the medial and lateral malleoli. The *Z*-axis of the rotation on the transverse plane revolves around the long axis of the tibia, perpendicular to the *Y*-axis. It intersects at the midpoint of the line connecting the medial and lateral malleoli. This point is the center of rotation of the ankle joint in the neutral position. During static standing, the *X*-axis of rotation on the coronal plane is perpendicular to the other two axes and passes through the center of rotation. To simulate static standing accurately, each model was subjected to a downward force of 300N (Anwar et al., 2017) applied vertically to the tibia and fibula, and the stress distribution on the ankle joint was recorded. Additionally, a rotational torque of 1.5 Nm (Zhang et al., 2022) was applied to the calcaneus to replicate internal and external rotation, supplementing the 300N static standing force. The talus rotation angle was recorded for further analysis. The corresponding ligamentous tissues were excised from the model to simulate an ankle joint with varying degrees of ligament damage. Based on preliminary experiment outcomes (Shoji et al., 2019; Sakakibara et al., 2020) and current clinical data (Mizrahi et al., 2018; Barini et al., 2021), emphasis was placed on the ATFL as the primary lateral ligament. The ATFL ligament was then paired with ruptures of the deltoid ligament to create compound ligament injury models. Consequently, the combined injury models were delineated as: lateral ligament injury + superficial deltoid ligament injury (ATFL + TNL; ATFL + TSL; ATFL + TCL; ATFL + sPTTL; ATFL + TNL + TSL + TCL + sPTTL) and lateral ligament injury + deep deltoid ligament injury (ATFL + ATTL; ATFL + dPTTL; ATFL + ATTL + dPTTL) and Complete rupture of all four superficial medial ligaments (TNL + TSL + TCL + sPTTL). In total, 11 distinct ankle models were established including intact ankle joints and isolated ATFL injuries.

## Result

### Validation of FE foot model

**FIGURE 4:**
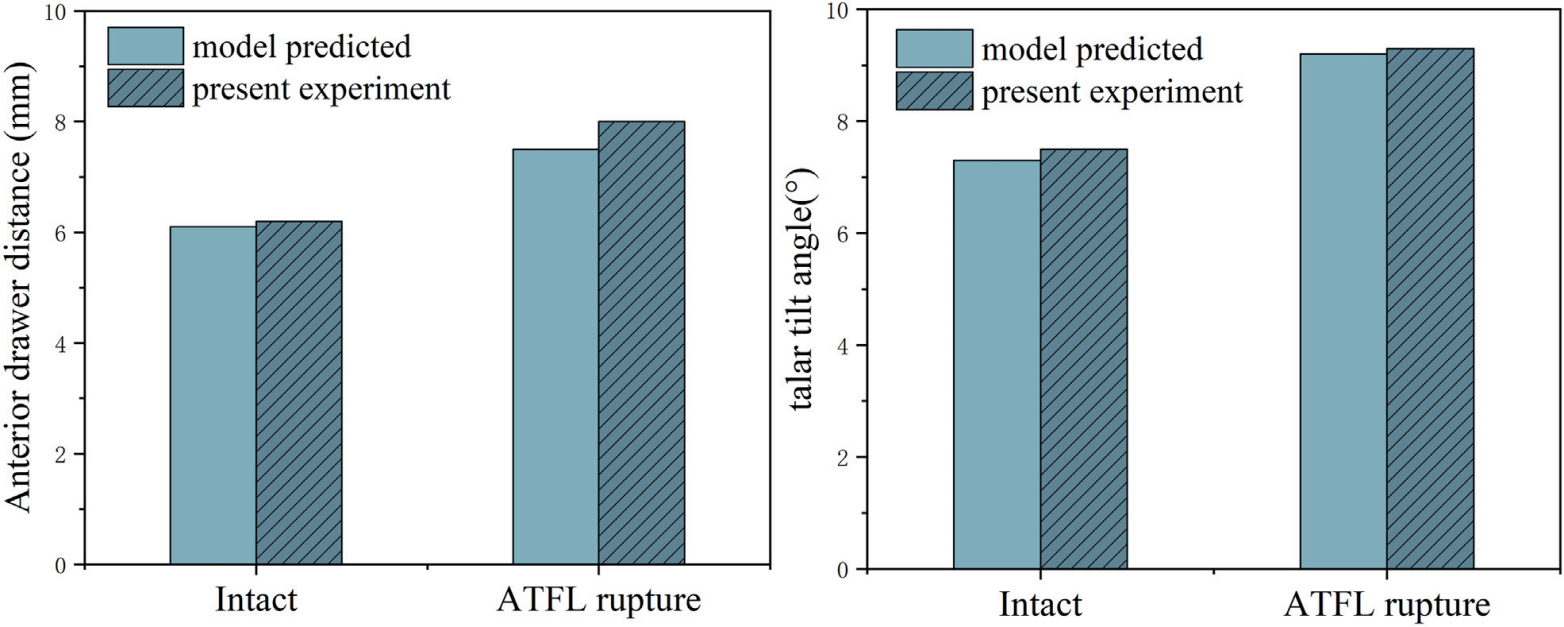
Anterior drawer distance and talus tilt angle in finite element model and cadaveric specimen biomechanical experiments.

Figure 4 presents the validation results of both the anterior drawer and talar tilt tests. The outcomes of the finite element (FE) models align well with the experimental data. When subjected to an anterior drawer force of 150N, the vertical distance between the posterior edges of the tibia and talus—before and after severing the ATFL—measures 6.2 mm and 8.0 mm, respectively. The FE models predicted values of 6.1 mm and 7.5 mm. Similarly, under a 150N lateral force, the angular displacement between the tibia and talus, pre and post-ATFL severance, was 7.3° and 9.2°, respectively, compared to the FE predictions of 7.5° and 9.3°. Notably, the discrepancies between the experimental and FE results were within a 6.5% margin.

Second, the validation results for the alignment of the various foot ligaments are shown in Supplementary Table S1. The FE model’s predictions of the ligament’s length, width, and thickness were highly consistent with the results of anatomical measurements, with maximum differences of 2.0 mm, 0.6 mm, and 0.2 mm, respectively, all within the range of previous research findings.

The validation results for the distances between the insertion points of each foot ligament and the corresponding bone markers are shown in Table 2. The FE model’s predictions of the ligament insertion points were highly consistent with the anatomical measurements, with a maximum difference of 1.1 mm, both within the range of prior research outcomes.

**TABLE 2:** Compared with the measurement results and literature, the FE model predicts the distance from the ligament insertion point to the relevant bony landmarks.

Ligaments	Insertion	Relevant osseous landmarks	Distances from relevant osseous landmarks		
			References data	Present measurement	FE predicted
ATFL	Fibular footprint	Inferior tip of lateral malleolus	13.8 (12.3–15.3)	14.2	14.5
	Talus footprint	Apex of lateral talar process	17.8 (16.3–19.3)	16.9	16.5
CFL	Fibular footprint	Inferior tip of lateral malleolus	5.3 (4.2–6.5)	6.0	6.2
	Calcaneus footprint	Posterior point of peroneal tubercle	16.3 (14.5–18.1)	14.8	14.2
PTFL	Fibular footprint	Inferior tip of lateral malleolus	4.8 (3.7–5.9)	6.2	5.7
	Talus footprint	Talar posterolateral tubercle	13.2 (11.5–14.9)	15.4	14.3
TNL	Tibia footprint	The distal center of the interfollicular groove	16.1 (14.7–17.7)	15.3	14.9
	Navicular footprint	Tuberosity of the navicular	9.7 (8.4–11)	11	10.4
TSL	Tibia footprint	The distal center of the interfollicular groove	13.1 (11.1–15.1)	12	11.4
	The insertion footprint	Of the spring ligaments posteroanterior distance	35%	37%	38%
TCL	Tibia footprint	The distal center of the interfollicular groove	6 (4.3–7.7)	5.8	5.3
	Calcaneus footprint	Posterior point of the sustenaculum tali	8 (7–9)	7.4	7.0
ATTL	Tibia footprint	The distal center of the interfollicular groove	11.1 (9.6–12.6)	12.0	11.5
	Talus footprint	The anteromedial corner of the trochlea	12.2 (11.1–13.4)	13.0	12.3
dPTTL	Tibia footprint	The distal center of the interfollicular groove	7.6 (6.7–8.5)	7.0	6.8
	Talus footprint	Posteromedial talar tubercle	17.8 (16.3–19.3)	12.7	12.3
sPTTL	Tibia footprint	The distal center of the interfollicular groove	3.5 (3.0–4.0)	3.9	4
	Talus footprint	Posteromedial talar tubercle	10.4 (8.9–11.9)	9.5	9.2

### The biomechanical simulation results of the intact and injured ankle joints

**FIGURE 5:**
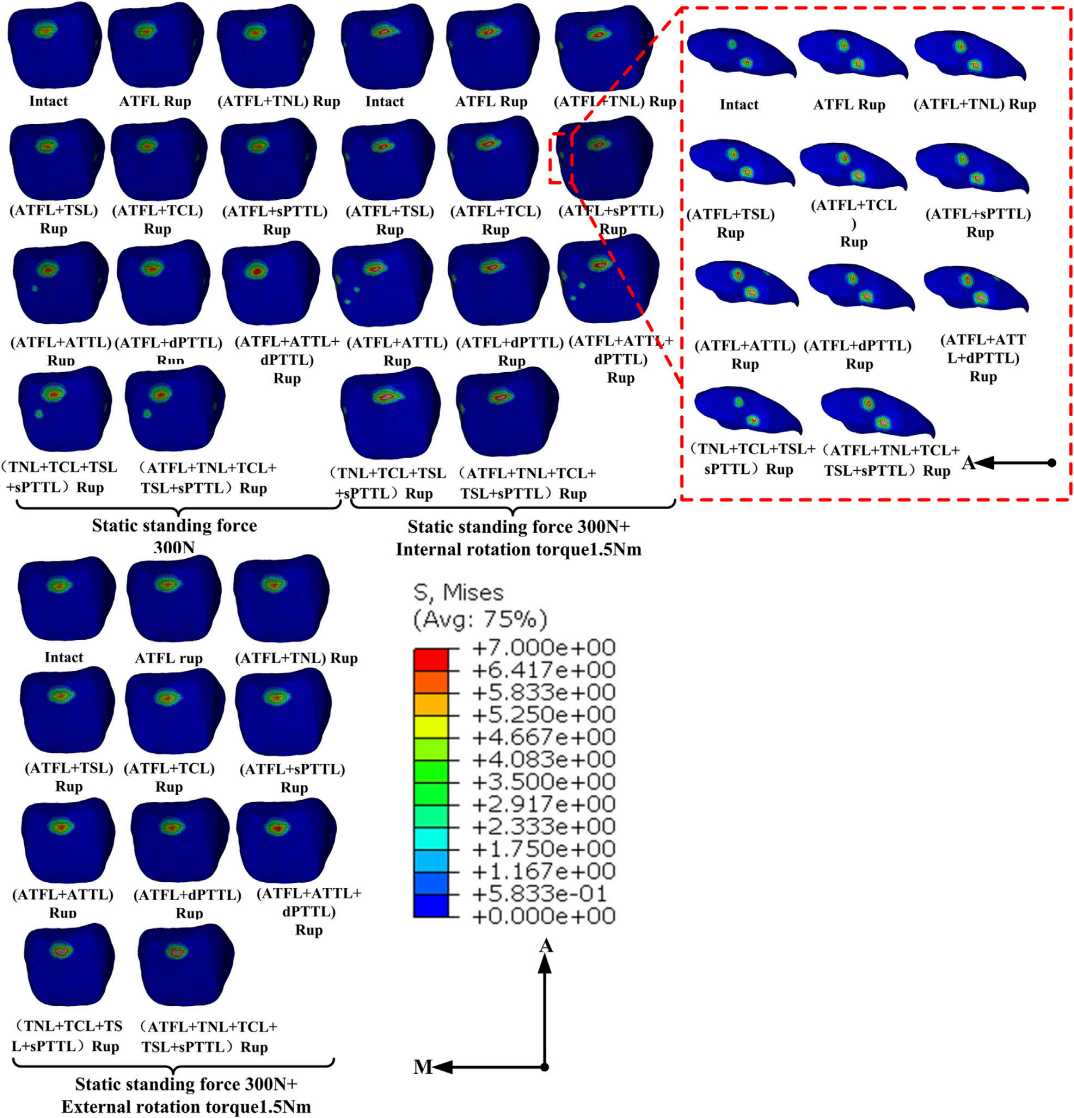
Distribution of von Mises stress (MPa) on the talus cartilage of ankle models with different injuries placed under various loading conditions.

The distribution of von Mises stress on the cartilage of the talocrural joint under various ligament rupture scenarios is shown in Figure 5. This figure shows that in a static standing position, stresses on the talar cartilage were primarily concentrated on the anterior and medial sides across the different injury models. When the ATFL and ATTL were ruptured concurrently, a new stress concentration area emerged in the middle of the talar articular surface, a region typically unstressed under normal conditions. This pattern is also evident in models featuring a complete rupture of the superficial DL4 bundle. Additionally, during static standing with internal rotation, the stress concentration extends considerably towards the medial side of the talar cartilage, beyond the surfaces of the talar dome. Moreover, in cases where the ATTL is among the compromised ligaments, internal rotation induces the formation of a novel contact region on the medial central portion of the upper surface of the talus cartilage. In static standing with external rotation, despite the similarity in stress concentration areas across the different ligament injury models, the stress on the talocrural joint cartilage intensifies when an ATFL rupture is coupled with a medial ligament rupture, as opposed to an isolated ATFL rupture.

**FIGURE 6:**
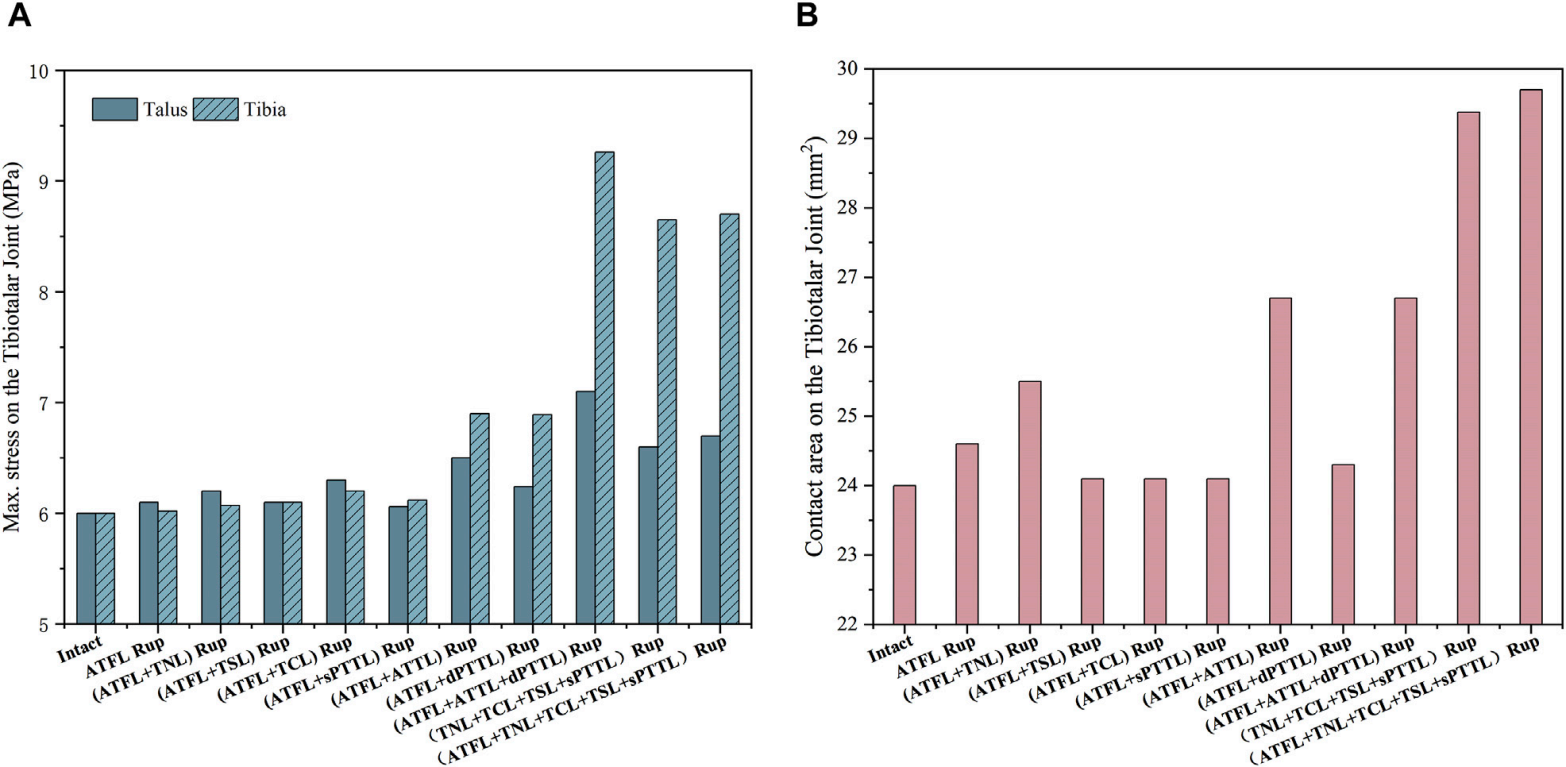
**(A)** the maximum von Mises stress on the tibia and talus cartilage with different injuries during static standing. **(B)** the contact area on the tibiotalar joint with different injuries during static standing.

Figure 6 illustrates the maximum von Mises stress experienced by the tibia and talus cartilage and the contact area in the tibiotalar joint under static standing conditions. The data shows that the maximum von Mises stress in the ankle joint with an isolated ATFL rupture increased by only about 0.3% and 1.6% in the tibia and talus cartilage, respectively, compared to an intact joint. A similar pattern was observed when an ATFL rupture was paired with a single superficial deltoid ligament strand rupture. However, the stress notably escalates with a rupture of the ATFL and ATTL, reaching a zenith when it coincides with an additional rupture of the dPTTL. Compared to the intact ankle joint, the stress increased by 18.3% in the talus and 54.3% in the tibial cartilage. Moreover, a rupture of the ATFL and the ATTL leads to an increased contact area in the talocrural joint, which is further enhanced when the ATFL and all four strands of the superficial medial ligaments rupture.

**FIGURE 7:**
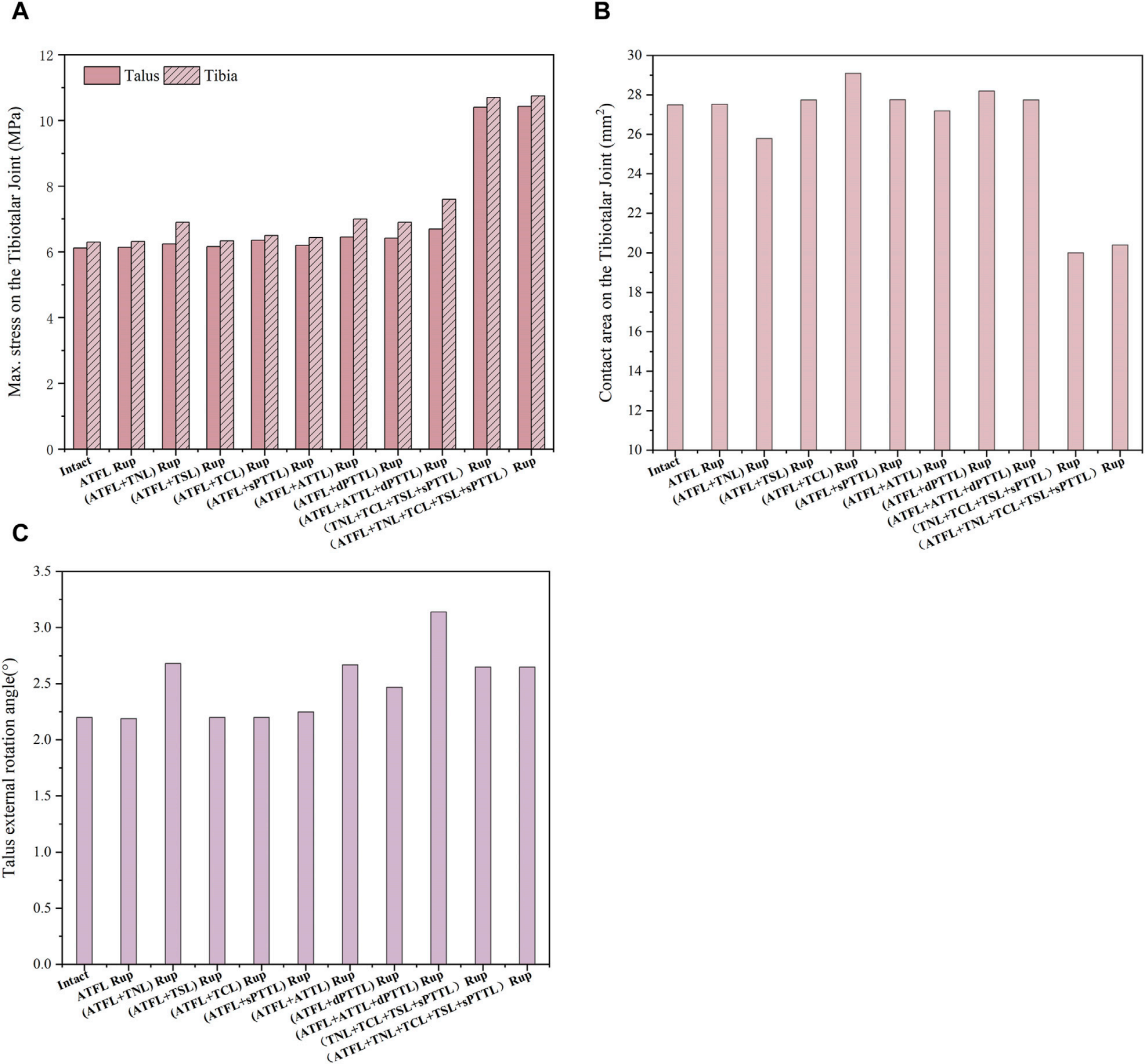
**(A)** the maximum von Mises stress on the tibia and talus cartilage with different injuries during static standing and external rotation. **(B)** The contact area on the tibiotalar joint with different injuries during static standing and external rotation. **(C)** The talus external rotation angle with different injuries during static standing and external rotation.

Figure 7 shows the maximum von Mises stress on the tibia and talus cartilage, the stress-induced contact area and the talar rotational angle under static standing and external rotation. The data shows that the maximum von Mises stress in the ankle joint with an isolated ATFL rupture increased by only about 0.3% in the tibia and talus cartilage compared to an intact joint. In contrast, the complete rupture of all four strands of the superficial deltoid ligament leads to a substantial increase in the peak stress: 69.8% increase in stress on the tibial cartilage, and 74.8% increase on the talus cartilage, in comparison to the intact joint. Furthermore, the total rupture of the deltoid ligament superficial strands reduced the contact area on the talocrural joint by 27.3%. Regarding the talus rotational angle, a combined rupture of the ATFL with either the superficial TNL or the deep ATTL increased the angle by 21.8%, relative to the intact ankle.

**FIGURE 8:**
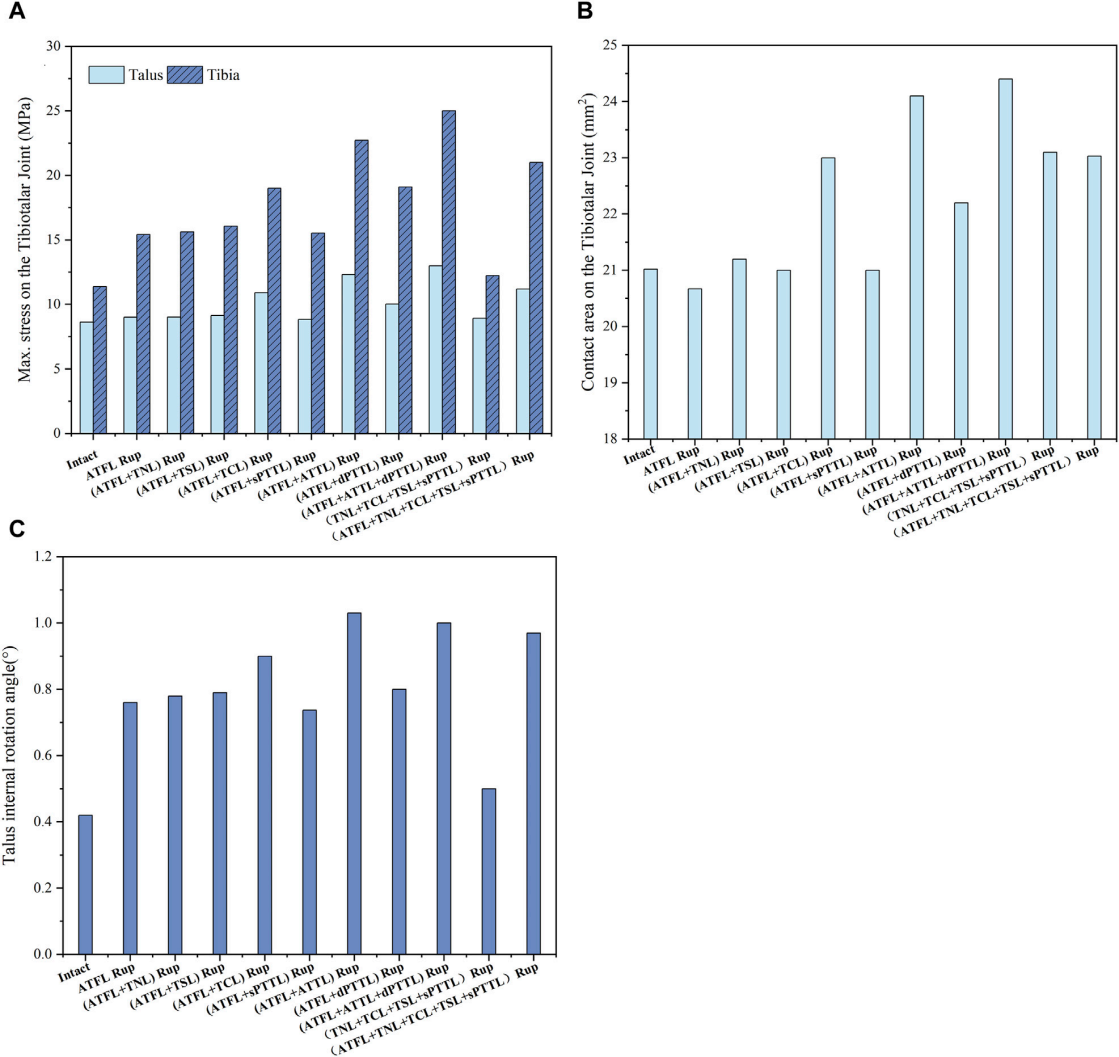
**(A)** the maximum von Mises stress on the tibia and talus cartilage with different injuries during static standing and internal rotation. **(B)** The contact area on the tibiotalar joint with different injuries during static standing and internal rotation. **(C)** The talus internal rotation angle with different injuries during static standing and internal rotation.

Figure 8 depicts the maximum von Mises stress on the tibia and talus cartilage during static standing and internal rotation, as well as the stress-induced contact area and the rotational angle of the talus. In the internal rotation position, all scenarios involving ATFL damage show increased maximum stress values of varying levels (greatest on the tibial side, ranging from 35.5% to 119.7%) and more pronounced increases when accompanied by ATTL rupture. Additionally, any rupture of the deep DL or superficial DL involving the TCL increases in contact area (ranging from 9.4% to 16.1%). Furthermore, compared to the intact ankle joint, an isolated ATFL rupture considerably increased the talus’s rotational angle by approximately 81%. When the ATFL and ATTL ruptured, the rotational angle of the talus further increased, reaching about 2.5 times that of the intact ankle joint.

## Discussion

The results of this research showed that an isolated ATFL rupture does not markedly alter the cartilage stress in an injured ankle joint relative to one with intact ligaments during the static standing and external rotation. However, when a multiband deltoid ligament (DL) continues to be injured, affecting either the superficial or deep layer, in a rotational ankle injury considerably alters the distribution of contact stress on the tibiotalar joint and increases the joint’s rotational angle under a specified torque. Conversely, when a single band of the DL is compromised, an ATTL injury significantly impacts the ankle’s stability of the normal mechanical architecture. Similarly, although an isolated ATFL injury increases the contact stress on the tibiotalar joint compared to an intact ankle joint during static standing and internal rotation condition, more severe joint stress abnormalities are observed when the DL continues to be injured.

Anatomical studies have demonstrated that the ATTL is typically smaller than the PTTL and is absent in some people (Campbell et al., 2014; Hintermann et al., 2014; Won et al., 2016), leading to the general assumption that the PTTL plays a more important role in ankle stability (Mengiardi et al., 2016). However, biomechanical testing on fresh ankle specimens has shown that the elastic modulus of the PTTL is approximately half of the ATTL (Siegler et al., 1988). In our research, we focused on scenarios where the ATFL was disrupted. We found that an ATTL rupture had a more pronounced impact on the mechanical structure of the ankle than a dPTTL rupture, except under conditions of external rotation torque. In such cases, the ATTL/dPTTL rupture predominantly affected the talus rotation angle. To our knowledge, this study is the first to simulate these specific conditions. In anatomical terms, the ATFL emerges from the fibula’s anterior-inferior segment and progresses inward towards the anterior region of the talus. Similarly, the ATTL originates from the tibia’s anterior-inferior portion, advancing to connect with the talus. These ligaments are situated in the anterior section of the ankle joint. In contrast, the PTTL arises from the posterior-inferior area of the tibia, descending to attach to the posterior region of the talus. Consequently, in instances of ATFL rupture, the ATTL may be essential in ensuring ankle joint stability, given its anterior placement. A study that sectioned the ligaments of 16 cadavers reported that the ATTL and intermediate tibiotalar ligaments control the ankle joint’s external rotation. Furthermore, these ligaments, in conjunction with the ATFL, regulate the internal rotation of the talus (Watanabe et al., 2012). A subsequent cadaveric study investigated the functionality of diverse deltoid ligaments under varying operational conditions (Gregersen et al., 2022). It was observed that the elongation and tension of the ATTL predominantly responded to rotational movements. In contrast, the length and tension of the dPTTL exhibit minimal sensitivity to such rotations. These findings corroborate our conclusion that the simultaneous rupture of the ATTL and ATFL substantially undermines the ankle joint’s rotational stability. In addition, previous study indicates significant variations in the length and tension of the dPTTL during ankle plantarflexion and dorsiflexion, implying that these positions may facilitate the observation of dPTTL rupture’s impact on the ankle joint’s mechanical structure (Gregersen et al., 2022). Nevertheless, the absence of tests on ankle plantarflexion and dorsiflexion in this study could explain the lack of significant contributions from the dPTTL that were detected.

Previous research has shown that chronic deltoid ligament (DL) injuries are often associated with cartilage lesions in the medial and anterior regions of the talus dome (Schäfer and Hintermann, 1996). MRI scans of patients with ankle instability have shown progressive degeneration in the talus dome’s posterior medial and lateral regions, even 3 years post-surgery (Hu et al., 2021). Some researchers have attributed the cartilage lesions to increased pressure on the joint surface (De Vries et al., 2005), while others argue that alterations in the distribution of the joint surface contact, referred to as joint contact patterns, are primarily responsible for the cartilage lesions (Alonso-Rasgado et al., 2017). Our study observed that the contact area of the tibiotalar joint was predominantly in the anterior medial region of the talar dome, with increased contact stress after ligament rupture. Additionally, new contact areas emerged in the medial posterior and central regions when the joint was subjected to a 300N vertical load with complete superficial DL rupture, with or without ATFL rupture, and when subjected to a 300N vertical load with 1.5 Nm internal rotation and with ATTL and ATFL rupture. These findings align closely with previous literature. Although our study did not simulate chronic ligament injuries or post-operative conditions, it underscores the importance of focusing on the medial region of the talar dome when treating DL injuries. Preventing articular facet cartilage degeneration post-surgery remains a significant challenge.

A previous study comparing various deltoid ligament (DL) reconstruction techniques (Wiltberger, Deland, Kitaoka, and Hintermann) through model simulations identified the Kitaoka procedure as the most effective for reducing ankle external rotation displacement (Xu et al., 2012). Among these techniques, the Kitaoka procedure uniquely positions the distal end of the reconstructed ligament anteriorly at the medial cuneiform (Kitaoka et al., 1998). Our study found that applying an external rotation torque led to a significant increase in talus external rotation in cases of ATTL/TNL rupture, particularly with single-band DL ruptures. These findings indicate that when treating rotational ankle instability (RAI) characterized by increased ankle external rotation, anteriorly relocating the distal insertion during DL reconstruction may help mitigate excessive external rotation.

This study has some limitations. 1) This study exclusively focused on static loading conditions, omitting considerations of ankle joint dorsiflexion and plantarflexion and the various loading environments associated with different daily activities. 2) The finite element model, derived from a single subject, may not accurately represent all patient groups. However, its advantage lies in the precise control over variables, permitting endless modifications of a specific factor without damaging the sample. Such methods are effectively used to enhance our understanding of the underlying biomechanics. 3) This research classified ligament conditions strictly as either ruptured or intact, deliberately omitting partial tears. Future studies will explore the biomechanics of the ankle joint after minor ligament tears leading to rotational instability.

## Conclusion

This study found that DL injury alters the distribution of contact stress on the tibiotalar joint and increases the talus rotation angle when subjected to rotational torque, which may lead to RAI. In addition to multiple-band DL injuries, it is also necessary to pay attention to single-band deep DL injuries, especially those that involve the ATTL.

## Data Availability

The original contributions presented in the study are included in the article/Supplementary Material, further inquiries can be directed to the corresponding authors.
