# Supplementary material for: Investigation into the effect of deltoid ligament injury on rotational ankle instability using a three-dimensional ankle finite element model
Source: Front Bioeng Biotechnol. 2024 May 1;12:1386401. doi: 10.3389/fbioe.2024.1386401 (PMC11094218; doi:10.3389/fbioe.2024.1386401)
Supplement: Supplementary file 1 [file Table1.docx]

Supplementary Material

Supplementary Table 1 Comparison of the ligament information predicted by finite element analysis with the results in the literature and the results of the current anatomical experiment.

|  |  | Length(mm) | Thickness(mm) | | | Width(mm) | | |
| --- | --- | --- | --- | --- | --- | --- | --- | --- |
| Ligaments |  | Middle | Proximal | Middle | Distal | Proximal | Middle | Distal |
| ATFL | Reference data | 15.5±7.7(Raheem and O'Brien, 2011)  20.17±3.4(Khawaji and Soames, 2015) | 1.15±0.28(Inchai et al., 2023) | 1.88±0.07(Peng et al., 2023)  1.01±0.35  1.06±0.32(Khawaji and Soames, 2015) | 0.81±0.29(Inchai et al., 2023) | 10.77±1.56(Taser et al., 2006)  5.21±1.52(Khawaji and Soames, 2015) | 6.75±2.89(Taser et al., 2006)  4.97±1.46(Khawaji and Soames, 2015) | 10.96±2.38(Taser et al., 2006)  4.59±1.41(Khawaji and Soames, 2015) |
|  | Present measurement | 11.3 | 1.4 | 1.5 | 1.3 | 7.4 | 6.8 | 7.2 |
|  | FE predicted | 11.8 | 1.2 | 1.5 | 1.2 | 7.5 | 7.1 | 7.3 |
| CFL | Reference data | 18.5±6.3(Raheem and O'Brien, 2011) | 1.43±0.49(Inchai et al., 2023) | 2.13±0.5(Dimmick et al., 2008)  1.11±0.37(Inchai et al., 2023) | 0.8±0.35(Inchai et al., 2023) | 7.19±2.23(Taser et al., 2006)  6.52±1.25(Inchai et al., 2023) | 4.68±1.34(Taser et al., 2006)  5.08±1.1(Inchai et al., 2023) | 9.68±1.73(Taser et al., 2006)  8.44±2.38(Inchai et al., 2023) |
|  | Present measurement | 20.2 | 0.9 | 1.0 | 0.7 | 5.3 | 4.0 | 5.7 |
|  | FE predicted | 18.39 | 0.8 | 0.8 | 0.6 | 5.0 | 4.5 | 6.0 |
| PTFL | Reference data | 21.66±4.84(Taser et al., 2006) | 2.13±0.63(Inchai et al., 2023) | 3.3±0.2(Stella et al., 2021)  1.84±0.51 | 0.96±0.48(Inchai et al., 2023) | 7.22±1.26(Inchai et al., 2023) | 5.55±1.25(Taser et al., 2006)  8.0±1.27 | 9.15±1.48(Inchai et al., 2023) |
|  | Present measurement | 11.5 | 0.9 | 0.7 | 0.7 | 6.7 | 6.4 | 7.7 |
|  | FE predicted | 11.6 | 0.8 | 0.8 | 0.6 | 6.4 | 6.7 | 8.0 |
| AITFL | Reference data | 15.46±4.22(Ebraheim et al., 2006) | - | 2.62±0.53(Ebraheim et al., 2006) | - | 4.92±1.21(Ebraheim et al., 2006) | 8.28±2.2(Ebraheim et al., 2006) | 3.76±0.52(Ebraheim et al., 2006) |
|  | Present measurement | 12.3 | 1.2 | 0.9 | 1.1 | 5.8 | 6.6 | 6.0 |
|  | FE predicted | 11.8 | 1.3 | 1.0 | 1.2 | 6.1 | 7.0 | 5.4 |
| PITFL | Reference data | - | - | 1.2±0.2(Stella et al., 2021) | - | - | 17.44±3.54(Ebraheim et al., 2006) | - |
|  | Present measurement | 13.8 | 0.8 | 0.8 | 0.6 | 12.4 | 11.8 | 10.5 |
|  | FE predicted | 14.2 | 0.7 | 0.7 | 0.6 | 13.0 | 12.0 | 11 |
| TNL | Reference data | 26.6±8.0(Won et al., 2016) | - | 0.8±0.3(Won et al., 2016) | - | 11±3.8(Milner and Soames, 1998)  5.2±2.3(Won et al., 2016) | 13.5±5.4(Milner and Soames, 1998)  7.3±3.3(Won et al., 2016) | 27.5±10.3(Milner and Soames, 1998)  25.5±14.6(Won et al., 2016) |
|  | Present measurement | 30.2 | 0.3 | 0.3 | 0.3 | 9.4 | 14.3 | 16.0 |
|  | FE predicted | 30.24 | 0.4 | 0.4 | 0.3 | 8.6 | 14.8 | 16.8 |
| TSL | Reference data | 18.5±6.3(Milner and Soames, 1998)  24±3.7(Won et al., 2016) | - | 1.6±0.6(Won et al., 2016) | - | 9.2±2.4(Hintermann et al., 2014)  8.0±2.3(Won et al., 2016) | 8.6±2.0(Hintermann et al., 2014)  7.0±1.8(Won et al., 2016) | 12.6±2.3(Hintermann et al., 2014)  9.7±2.7(Won et al., 2016) |
|  | Present measurement | 25 | 0.3 | 0.7 | 0.6 | 4.7 | 5.6 | 8.2 |
|  | FE predicted | 24.4 | 0.4 | 0.8 | 0.7 | 4.1 | 5.5 | 8.7 |
| TCL | Reference data | 18.0±7.7(Milner and Soames, 1998)  23.7±3.9(Won et al., 2016) | - | 1.6±0.7(Won et al., 2016) | - | 8.1±2.1(Hintermann et al., 2014)  5.5±2.1(Won et al., 2016) | 8.4±1.9(Hintermann et al., 2014)  5.0±2.0(Won et al., 2016) | 11.1±1.7(Hintermann et al., 2014)  5.7±2.2(Won et al., 2016) |
|  | Present measurement | 20.9 | 0.6 | 0.7 | 0.7 | 4.8 | 4.7 | 4.7 |
|  | FE predicted | 19.9 | 0.7 | 0.9 | 0.8 | 4.5 | 4.8 | 5.0 |
| ATTL | Reference data | 11.4±2.7(Won et al., 2016)  8.8±2.3 | - | 1.6±0.7(Won et al., 2016) | - | 7.9±2.8(Hintermann et al., 2014)  3.6±1.5(Won et al., 2016) | 7.6±2.7(Hintermann et al., 2014)  3.2±1.2(Won et al., 2016) | 7.9±2.8(Hintermann et al., 2014)  4.3±1.8(Won et al., 2016) |
|  | Present measurement | 10.9 | 1.1 | 1.2 | 1.3 | 4.7 | 5.0 | 5.7 |
|  | FE predicted | 11.1 | 1.3 | 1.2 | 1.5 | 4.5 | 5.2 | 5.6 |
| dPTTL | Reference data | 14.5±2.5(Won et al., 2016)  9.6±2.6 | - | 7.5±1.5(Won et al., 2016) | - | 11.2±2.3(Hintermann et al., 2014)  10.6±1.5(Won et al., 2016) | 10.3±2.4(Hintermann et al., 2014)  10.3±1.4(Won et al., 2016) | 10.9±2.4(Hintermann et al., 2014)  11.2±1.9(Won et al., 2016) |
|  | Present measurement | 10.9 | 2.1 | 2.2 | 2.1 | 7.0 | 7.1 | 7.1 |
|  | FE predicted | 12.3 | 1.9 | 2.0 | 1.9 | 7.5 | 7.6 | 7.6 |
| sPTTL | Reference data | 21±3.8 | - | - | - | - | - | - |
|  | Present measurement | 16.9 | 0.5 | 0.5 | 0.5 | 4.6 | 5.2 | 4.5 |
|  | FE predicted | 17.3 | 0.6 | 0.5 | 0.6 | 4.3 | 4.8 | 4.7 |
